# Comparison of Negative Pressure Wound Therapy With and Without Instillation of Saline in the Management of Infected Wounds

**DOI:** 10.7759/cureus.9047

**Published:** 2020-07-07

**Authors:** Paul J Kim, Ronald Silverman, Christopher E Attinger, Leah Griffin

**Affiliations:** 1 Plastic Surgery, University of Texas Southwestern Medical Center, Dallas, USA; 2 Plastic Surgery, University of Maryland, Baltimore, USA; 3 Medical Solutions Division, 3M, San Antonio, USA; 4 Plastic Surgery, MedStar Georgetown University Hospital, Washington, DC, USA; 5 Health Economics and Reimbursement, 3M, San Antonio, USA

**Keywords:** negative pressure wound therapy, instillation, chronic wounds, wound healing

## Abstract

Background

Negative pressure wound therapy (NPWT) with instillation and dwell time (NPWTi-d) includes periodic instillation of topical solution into the wound followed by a negative pressure. Our objective was to evaluate potential differences in wound outcomes in patients receiving NPWT and those receiving NPWTi-d using saline.

Methods

An analysis was performed using two previously published independent studies from a single investigator and hospital to compare patient characteristics and clinical outcomes of infected wounds from 74 NPWT-treated patients with 42 NPWTi-d-treated patients.

Results

Patient demographics and comorbidities, wound etiologies, and anatomical locations of wounds were similar between groups, although a significantly higher percentage of NPWT-treated patients had end-stage renal disease (P = 0.0119). Compared with patients treated with standard NPWT, NPWTi-d-treated patients had a significantly lower number of operations (P = 0.0048), shorter length of hospital stay (P = 0.0443), shorter time to final surgical procedure (P = 0.0001), higher percentage of closed wounds (P = 0.0004), and a higher percentage of wounds that remained closed at one month (P = 0.0001).

Conclusions

The results of this analysis suggest that management of infected wounds with NPWTi-d using saline leads to favorable wound outcomes when compared to those managed with NPWT.

## Introduction

Increased patient morbidity and mortality, length of hospital stay, and costs are associated with infection in both acute and chronic wounds [1]. Wound infection management strategies include the use of antibiotics and the removal of infectious materials. While numerous advanced wound care products assist in the management of wound infection, negative pressure wound therapy (NPWT) utilizes negative pressure to remove exudate and infectious materials from wounds. The resulting negative pressure draws wound edges together and promotes angiogenesis and granulation tissue formation in the wound bed [2-5].

NPWT has evolved to include the periodic instillation of topical wound solutions directly over the wound bed, followed by removal using negative pressure. This NPWT with instillation and dwell time (NPWTi-d) utilizes the same properties of NPWT with the added benefit of wound cleansing [6]. NPWTi-d has been reported to promote wound cleansing, granulation tissue development, and healing in wounds that did not respond to traditional NPWT [7-9]. The comparative effectiveness of NPWTi-d using normal saline, a recommended first-line NPWTi-d solution, versus standard NPWT has not been adequately assessed in previous studies [10-12]. Our objective was to evaluate potential differences in wound outcomes in patients at an institution receiving NPWT and those receiving NPWTi-d with saline.

## Materials and methods

An analysis was performed using two independent previously published studies from a single investigator and hospital to compare patient characteristics and clinical outcomes of infected wounds from 74 NPWT-treated patients from the article’s retrospective control cohort (control group) with 42 NPWTi-d-treated patients from the article’s per protocol population (study group) [13,14]. As previously described, all patients underwent excisional debridement in the operating room and received parenteral or oral antibiotics [13,14]. The control group received continuous negative pressure at -125 mmHg using NPWT (INFOV.A.C.™ Therapy System, KCI, San Antonio, TX) [13]. The study group received NPWTi-d (V.A.C. VERAFLO™ Therapy, KCI, San Antonio, TX) with instillation of 0.9% saline with a dwell time of 20 minutes followed by two hours of negative pressure (-125 mmHg) [14]. Outcomes assessed included the number of operations, time to final surgery, length of hospital stay, wound closure, and percentage of wounds that remained closed at one month. Wound closure was defined as coverage of wound through delayed primary closure, skin graft, or flap. Statistical significance was determined using a t-test for continuous variables or Fisher’s exact test for categorical values. Results were considered statistically significant at a P-value ≤0.05.

## Results

The mean age of patients in the control group (n = 74) and the study group (n = 42) was 58.0 ± 13.0 years and 60.7 ± 15.1 years, respectively (Table 1). Patient demographics, comorbidities, wound etiologies, and anatomical locations of wounds were similar between groups, although a significantly higher percentage of NPWT-treated patients had end-stage renal disease (P = 0.0119) (Tables 1, 2).

**Table 1:** Patient demographics and comorbidities BMI = body mass index; ESRD = end-stage renal disease; PVD = peripheral vascular disease; SD = standard deviation

Characteristics	Control Group (n = 74)	Study Group (n = 42)	P-value
Age, years (mean ± SD)	58.0 ± 13.0	60.7 ± 15.1	0.3202
BMI, kg/m^2^ (mean ± SD)	32 ± 9.1	29.1 ± 8.2	0.0913
Gender, n (%)			0.2429
Male	38 (51.0)	27 (64.0)	
Female	36 (49.0)	15 (36.0)	
Race, n (%)			0.0995
African American	21 (28.0)	19 (51.4)	
Caucasian	39 (53.0)	17 (45.9)	
Hispanic	2 (6.0)	0 (0)	
Asian	1 (3.0)	1 (2.7)	
Other race	6 (8.0)	0 (0)	
Comorbidities, n (%)			
ESRD	22 (30.0)	4 (9.5)	0.0119
PVD	27 (36.0)	9 (21.4)	0.1004
History of cancer	6 (8.0)	6 (14.3)	0.3477

**Table 2:** Wound type and anatomical location AKA = above-knee amputation; BKA = below-knee amputation; TMA = transmetatarsal amputation

Characteristic	Control Group (n = 74)	Study Group (n = 42)
Wound type, n (%)		
Ischemic	17 (23.0)	6 (14.3)
Neuropathic	16 (22.0)	14 (33.3)
Decubitus	16 (22.0)	4 (9.5)
Surgical	17 (23.0)	13 (31)
Venous insufficiency	3 (4.0)	2 (4.8)
Traumatic	4 (5.0)	1 (2.4)
Other	3 (4.0)	1 (4.8)
Anatomical location, n (%)		
Forefoot	12 (16.0)	10 (23.8)
Midfoot	12 (16.0)	2 (4.8)
Hindfoot	22 (30.0)	3 (7.1)
TMA site	1 (1.0)	6 (14.3)
Ankle	7 (9.0)	7 (16.7)
Lower leg	7 (9.0)	5 (11.9)
BKA/AKA	1 (1.0)	1 (2.4)
Knee	1 (1.0)	3 (7.1)
Thigh	3 (4.0)	0 (0)
Back/buttock	2 (3.0)	3 (7.1)
Abdomen	5 (7.0)	2 (4.8)
Arm	1 (1.0)	0 (0)

Compared with the control group patients, the study group patients had a significantly lower number of operations (P = 0.0048), shorter length of hospital stay (P = 0.0443), and shorter time to final surgical procedure (P = 0.0001). Additionally, higher percentage of closed wounds (P = 0.0004) and higher percentage of wounds that remained closed at one month (P = 0.0001) were observed in the study group (Table 3).

**Table 3:** Clinical outcomes SD = standard deviation

Characteristic	Control Group (n = 74)	Study Group (n = 42)	P-value
Number of operations (mean ± SD)	3.0 ± 0.9	2.5 ± 0.9	0.0048
Length of hospital stay, days (mean ± SD)	14.9 ± 9.2	11.7 ± 6.0	0.0443
Time to final procedure, days (mean ± SD)	9.2 ± 5.2	5.6 ± 3.6	0.0001
Wound closure/coverage, n (%)	46 (62)	39 (92.9)	0.0004
Wounds remained closed at one month, n (%)	28 (37.8)	32 (82.1)	0.0001

## Discussion

Wound infection can create a barrier to healing and increase patient morbidity and healthcare costs [1]. While treatment includes the use of bacteria-specific antibiotics, advanced wound therapies play an important role in wound management during treatment. NPWT can help manage wounds through the use of negative pressure to remove exudate and infectious materials. NPWT use in infected wounds has been reported as safe for patients [15,16]. Product advancements have led to the addition of wound cleansing to NPWT, which may provide an additional wound management option to patients with infected wounds. This study examined differences in wound outcomes in patients with infected wounds at one institution receiving either NPWT or NPWTi-d using saline.

NPWT uses macrostrain and microstrain resulting from negative pressure to draw wound edges together, remove infectious materials and exudate, reduce edema, and promote angiogenesis and granulation tissue formation in the wound bed [2-5]. NPWTi-d utilizes these same properties with the added benefit of wound cleansing with the instillation of topical wound solutions [6]. However, while the clinical benefit of NPWTi-d use has been shown, limited published evidence exists for NPWTi-d use in infected wounds. 

In this study, significantly lower number of operations, shorter length of hospital stay, shortened time to final procedure, higher percentage of closed wounds, and higher percentage of wounds that remained closed at the one-month follow-up visit were reported in the NPWTi-d group. These results are similar to those reported by Gabriel et al. and Omar et al. [11,12]. However, patients received either saline or a polyhexanide instillation solution in the Gabriel et al. study. Additionally, while a shorter hospital stay and time to wound closure were observed in the NPWTi-d group in the Omar et al. study, these were not statistically significant compared to the NPWT group [12]. The results of this analysis suggest that management of infected wounds with NPWTi-d using saline leads to favorable wound outcomes when compared to those managed with NPWT.

The retrospective nature and the analysis of only two previously published studies are limitations to this work. Limited data exist for the use of NPWTi-d in infected wounds [13,14]. The publications that were available for comparison used polyhexanide and saline instillation solutions with limited numbers of patients in each. Instead of a meta-analysis, we opted to assess patients treated by one clinician at one hospital using to provide a more direct comparison. Caution should be used when interpreting the conclusions of this study due to the limited scope of the analysis. Future, large-scale, controlled cohort studies are warranted to further assess the potential benefits associated with NPWTi-d use in the management of infected wounds.

## Conclusions

The results indicate that wound cleansing combined with NPWT may provide an additional clinical benefit in the management of infected wounds. However, due to the limited analysis, conclusions should be interpreted with caution. Future studies assessing the potential benefits of NPWTi-d use in the management of infected wounds are necessary.

## References

[1] Zakhary SA, Davey C, Bari R (2017). The development and content validation of a multidisciplinary, evidence-based wound infection prevention and treatment guideline. Ostomy Wound Manage.

[2] McNulty A, Spranger I, Courage J, Green J, Wilkes R, Rycerz A (2010). The consistent delivery of negative pressure to wounds using reticulated, open cell foam and regulated pressure feedback. Wounds.

[3] Wilkes R, Zhao Y, Cunningham K, Kieswetter K, Haridas B (2009). 3D strain measurement in soft tissue: demonstration of a novel inverse finite element model algorithm on microCT images of a tissue phantom exposed to negative pressure wound therapy. J Mech Behav Biomed Mater.

[4] Saxena V, Hwang CW, Huang S, Eichbaum Q, Ingber D, Orgill DP (2004). Vacuum-assisted closure: microdeformations of wounds and cell proliferation. Plast Reconstr Surg.

[5] Orgill DP, Bayer LR (2013). Negative pressure wound therapy: past, present and future. Int Wound J.

[6] Kim PJ, Attinger CE, Constantine T (2020). Negative pressure wound therapy with instillation: international consensus guidelines update. Int Wound J.

[7] Gabriel A (2012). Integrated negative pressure wound therapy system with volumetric automated fluid instillation in wounds at risk for compromised healing. Int Wound J.

[8] Fluieraru S, Bekara F, Naud M, Herlin C, Faure C, Trial C, Téot L (2013). Sterile-water negative pressure instillation therapy for complex wounds and NPWT failures. J Wound Care.

[9] Brinkert D, Ali M, Naud M, Maire N, Trial C, Téot L (2013). Negative pressure wound therapy with saline instillation: 131 patient case series. Int Wound J.

[10] Kim PJ, Attinger CE, Crist BD (2015). Negative pressure wound therapy with instillation: review of evidence and recommendations. Wounds.

[11] Gabriel A, Kahn K, Karmy-Jones R (2014). Use of negative pressure wound therapy with automated, volumetric instillation for the treatment of extremity and trunk wounds: clinical outcomes and potential cost-effectiveness. Eplasty.

[12] Omar M, Gathen M, Liodakis E, Suero EM, Krettek C, Zeckey C, Petri M (2016). A comparative study of negative pressure wound therapy with and without instillation of saline on wound healing. J Wound Care.

[13] Kim PJ, Attinger CE, Steinberg JS (2014). The impact of negative-pressure wound therapy with instillation compared with standard negative-pressure wound therapy: a retrospective, historical, cohort, controlled study. Plast Reconstr Surg.

[14] Kim PJ, Attinger CE, Oliver N, Garwood C, Evans KK, Steinberg JS, Lavery LA (2015). Comparison of outcomes for normal saline and an antiseptic solution for negative-pressure wound therapy with instillation. Plast Reconstr Surg.

[15] Shweiki E, Gallagher KE (2013). Negative pressure wound therapy in acute, contaminated wounds: documenting its safety and efficacy to support current global practice. Int Wound J.

[16] Lo Torto F, Ruggiero M, Parisi P, Borab Z, Sergi M, Carlesimo B (2017). The effectiveness of negative pressure therapy on infected wounds: preliminary results. Int Wound J.

